# Development of a Mobile App for Ecological Momentary Assessment of Circadian Data: Design Considerations and Usability Testing

**DOI:** 10.2196/26297

**Published:** 2021-07-23

**Authors:** Thomas B Woolf, Attia Goheer, Katherine Holzhauer, Jonathan Martinez, Janelle W Coughlin, Lindsay Martin, Di Zhao, Shanshan Song, Yanif Ahmad, Kostiantyn Sokolinskyi, Tetyana Remayeva, Jeanne M Clark, Wendy Bennett, Harold Lehmann

**Affiliations:** 1 Department of Physiology Johns Hopkins University School of Medicine Baltimore, MD United States; 2 Department of Health Policy and Management Johns Hopkins University Bloomberg School of Public Health Baltimore, MD United States; 3 Division of General Internal Medicine Johns Hopkins University School of Medicine Baltimore, MD United States; 4 Department of Psychiatry and Behavioral Sciences Johns Hopkins University School of Medicine Baltimore, MD United States; 5 Department of Epidemiology Johns Hopkins University Bloomberg School of Public Health Baltimore, MD United States; 6 Division of Health Sciences Informatics Johns Hopkins University School of Medicine Baltimore, MD United States; 7 Department of Computer Science Johns Hopkins University Baltimore, MD United States; 8 SROST Toronto, ON Canada

**Keywords:** mhealth, circadian, sleep, ecological momentary assessment, timing of eating, mobile applications, habits, body weight, surveys and questionnaires

## Abstract

**Background:**

Collecting data on daily habits across a population of individuals is challenging. Mobile-based circadian ecological momentary assessment (cEMA) is a powerful frame for observing the impact of daily living on long-term health.

**Objective:**

In this paper, we (1) describe the design, testing, and rationale for specifications of a mobile-based cEMA app to collect timing of eating and sleeping data and (2) compare cEMA and survey data collected as part of a 6-month observational cohort study. The ultimate goal of this paper is to summarize our experience and lessons learned with the Daily24 mobile app and to highlight the pros and cons of this data collection modality.

**Methods:**

Design specifications for the Daily24 app were drafted by the study team based on the research questions and target audience for the cohort study. The associated backend was optimized to provide real-time data to the study team for participant monitoring and engagement. An external 8-member advisory board was consulted throughout the development process, and additional test users recruited as part of a qualitative study provided feedback through in-depth interviews.

**Results:**

After ≥4 days of at-home use, 37 qualitative study participants provided feedback on the app. The app generally received positive feedback from test users for being fast and easy to use. Test users identified several bugs and areas where modifications were necessary to in-app text and instructions and also provided feedback on the engagement strategy. Data collected through the mobile app captured more variability in eating windows than data collected through a one-time survey, though at a significant cost.

**Conclusions:**

Researchers should consider the potential uses of a mobile app beyond the initial data collection when deciding whether the time and monetary expenditure are advisable for their situation and goals.

## Introduction

Establishing the impact of daily habits on long-term health is an important but challenging goal [1-3]. It is well-established that poor daily habits, continued over many years, can lead to adverse health outcomes [4-6]. Less clear is how to collect individuals' daily habits in a way that encourages accurate and comprehensive reporting. We present our experience in developing a research environment for collecting data to explore associations between sleep and eating patterns (the daily habits) and weight (the health outcome) [4,7-10].

This type of app falls into the category of research tools used for ecological momentary assessment (EMA) [11-14]. Since the behaviors are daily and repetitive, we use the term “circadian EMA” (cEMA) and label all similar apps as a particular cEMA app. In general, all EMA apps address circadian problems: For example, how does a participant’s mood change over multiple days [15] and how much exercise has a participant been getting and does that correlate with sleep [16]? These are both examples of measures that are changing (or have the potential to change) within and across days, and so finding the correlations between daily habits and some measure of long-term change is common to much of the EMA literature. Many EMA apps have been developed with different research foci than ours. Other intermittent behaviors, such as exercise and alcohol or drug use, call for a different app design.

This paper sets out the requirements and design choices for mobile-based cEMAs that we faced during the creation of the Daily24 app. We note that others have addressed requirements for EMA, and we endeavor to add our experience without repeating previous observations [14,17-32]. The environment we describe consists of a user-facing smartphone-based app (Daily24), a back-end server to receive the data, and a research dashboard that enables management of the research process. We highlight the new challenges and opportunities provided through the ability to interact with study participants in real time for a period of months. We describe our design, testing, and key revisions to the app and the associated back end and present our design choices for automated messaging to participants and our real-time dashboards to monitor participant engagement. We then summarize the feedback collected through our user testing protocol and present a comparison of data collected through the app with recall data collected through a one-time, web-based survey. We end with additional considerations when deciding whether to use a mobile app in other research settings.

## Methods

### Parent Study Design

**Figure 1:**
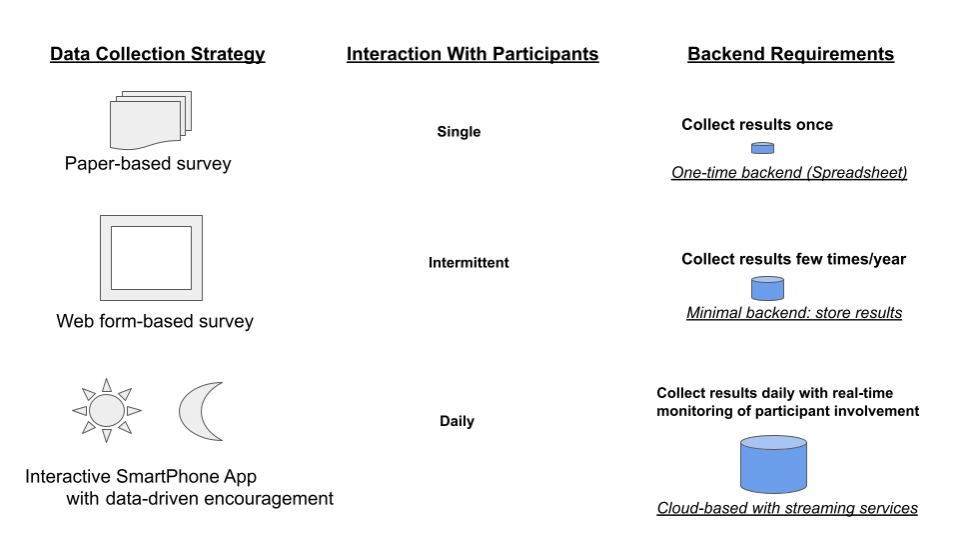
Comparing paper-based, web form–based, and smartphone app–based survey approaches. Note that paper-based surveys can be sent to more than one population or re-used, but often require manual data entry.

The overall study for which the mobile app was developed aimed to electronically recruit 1000 app users from the outpatient population at 3 major medical centers. Participants were expected to use the app periodically over the 6-month study period, specifically daily during the first 4 weeks (referred to as the “Power28”), then for 1 week in each of the remaining 5 months (the “PowerWeeks”). Participants were also asked to complete periodic online surveys and to grant permission to access their electronic medical record (EMR) data. A mobile app was considered the optimal data collection modality for this study given the long duration and emphasis on capturing daily variability (Figure 1). Inclusion criteria included being 18 years of age or older, English-speaking, and a patient of the sponsoring institutions and with access to a smartphone with data or Wi-Fi connection to transmit app data.

### User Testing Study Design

**Figure 2:**
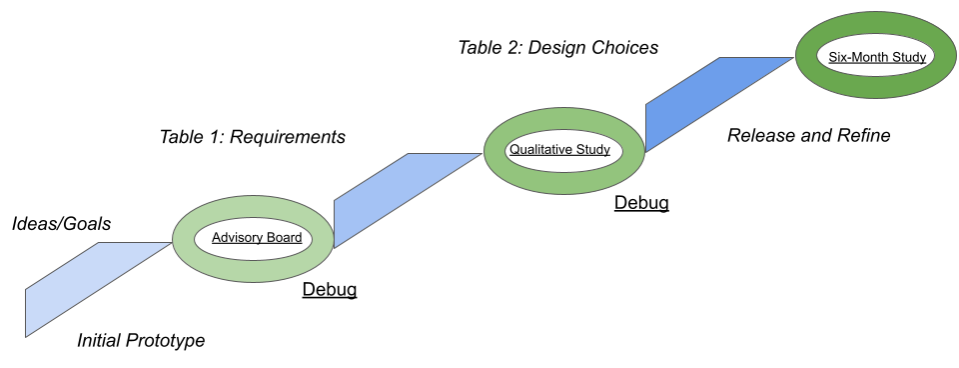
The agile development methodology used to stage the release and use of the app for the parent study.

The study team recognized the essential need to gather input from test users outside of the team to inform acceptability and usability of the app throughout the development process (Figure 2). During the initial development process, an advisory board of 8 individuals identified as patient advocates for the population we hoped to recruit from was assembled to provide feedback on design, usability, and ease of use. The advisory board was convened 3 times during the app development period. The first was during the design process to share the goals of the app and solicit feedback on the visual presentation. Board members then downloaded the first minimal version of the app and provided initial reactions at the next meeting, with additional feedback provided by phone after 2 weeks of use. At the final meeting, members were consulted about the recruitment and consent strategies for the cohort study, which was atypical in being conducted entirely online with no person-to-person interaction.

After the initial app development was complete, we conducted a qualitative study to elicit feedback on the usability of the app from target potential users and also to test the back-end linkages between app data and the individual participant’s EMR. Qualitative study participants were asked to complete a baseline online questionnaire, then download and use the test app for at least 4 days before meeting with a member of the study team for a 1-hour in-depth interview [33]. Inclusion criteria were the same as for the parent study, with the exception that participants had to be patients of the sponsoring institution.

### Development of the Mobile App Front End

Based on participant characteristics from previous studies enrolling electronically from a similar population, we created a persona of the demographic most likely to enroll for the purposes of designing the app: a woman in her mid-50s, with at least a high-school education, and with an interest in contributing research data for the greater good since there was no monetary compensation provided for participation. We did not expect all participants to be health-conscious or tech-savvy. We also imagined our participants as being concerned about their weight, but not self-managing multiple other chronic conditions or severe acute illness.

We wanted to enable a design that could be reused for other similar studies and that could scale up or down depending on the study size and population. We specifically conducted a multicenter study to establish the generalizability of both our app and our results. Additionally, to avoid selection bias implicit in limiting to a single operating system and to accommodate as many participants as possible, we developed for both iOS and Android using React Native [19], a JavaScript-based, cross-platform development environment. This platform choice brought with it some slower responses within the app and also meant that some user interface items that users on each platform take for granted were not readily available.

The most basic requirement for the mobile app was that the data could be collected on a daily basis, as part of the user’s regular routine. For Daily24, the behaviors of interest were sleeping and eating, including wake time, sleep time, start and end time of eating, and an estimate of amount eaten. Our goal was to design the interface to be as simple as possible and to encourage minimal time investment for each participant in the daily logging of information. We had developed an earlier version (Metabolic Compass) of our final app that drew praise from about 50 users for its ability to present data back to participants; however, for this study, we wanted to limit the data shared back with users to keep from influencing their daily patterns.

An additional tradeoff and design decision was to forgo collecting detailed dietary data, choosing instead to collect estimated size of the eating occasion. There are many currently popular apps (for example, MyFitnessPal, MyPlate) that capture caloric information. However, the entry of detailed dietary data takes significant time and would add complexity to the simple interface we wanted and would overwhelm the user’s altruism in providing data for research purposes. Additionally, the support required for the large lookup tables for calorie management was beyond both our budget and our developer team. We elected to include 6 broad categories of eating episodes: large, medium, and small meals; large and small snacks; and drinks (except water) without food.

**Figure 3:**
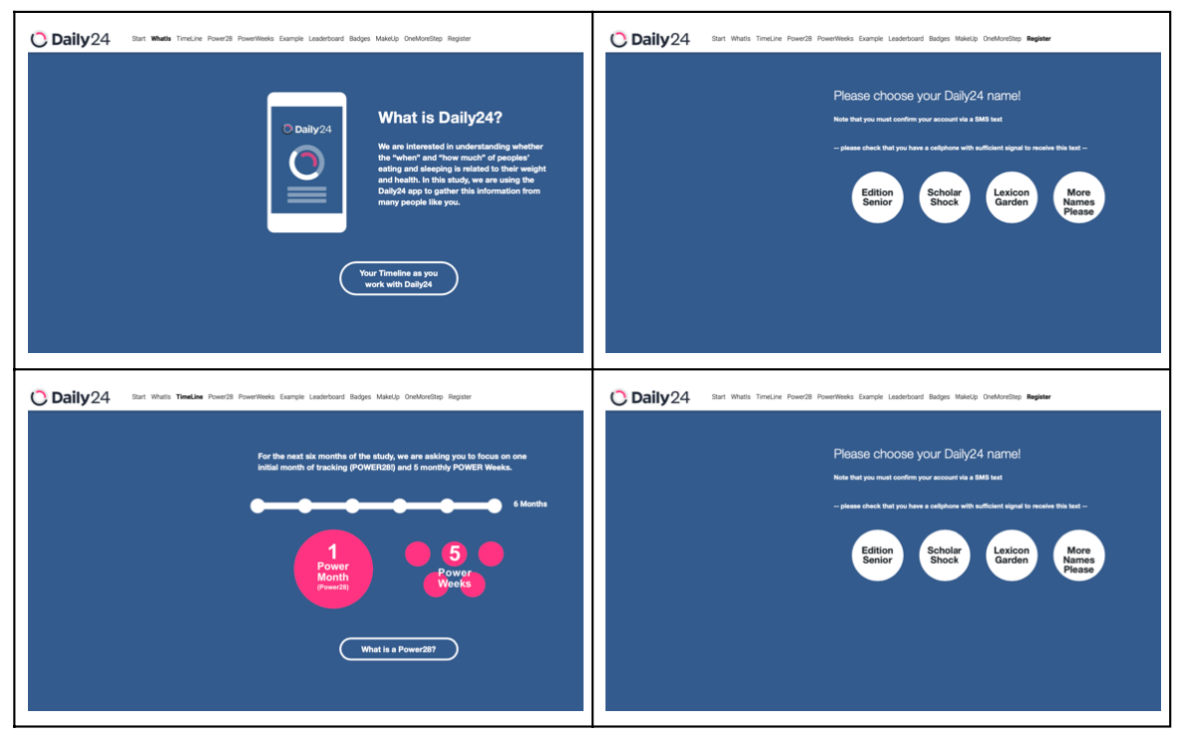
Daily24 registration screens.

**Figure 4:**
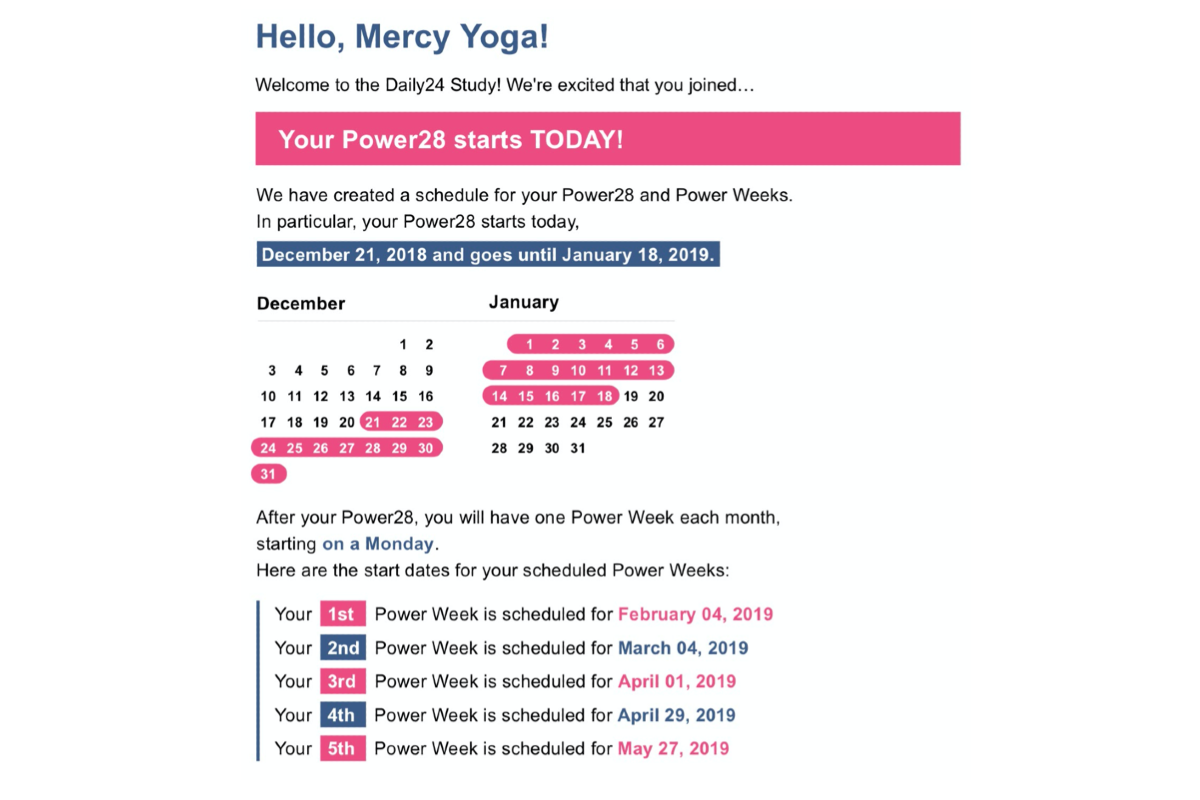
Examples of SMS and emails from a larger set of Amazon Web Services step function–controlled messages to participants.

Our budget did not permit the study coordination team to have frequent interactions with all participants and to encourage involvement with a high-touch modality. Therefore, we designed automated reminders and real-time dashboards to let our study coordination team identify those participants most in need of encouragement to stay actively involved. Users received reminders at sign-up and during their POWER28 and Power Weeks. Examples of the initial signup email, reminders, and update emails regarding data contributions are shown in Figures 3 and 4.

Given the research context, we designed the app to protect participant privacy and to encourage competition between participants without any sharing of identifiable information. To create usernames, we tested, refined, and ultimately used 2 large tables of uplifting and nonobjectionable adjectives, nouns, and verbs to create names that users could select and identify with, without having to directly craft the names themselves. Example usernames like “YoungWinter” were fully separated from identifiable information. We used a universally unique identifier (for example “7beb5648a909a16fa39281e0ee1b564b”) to link our data used for messaging with the data being collected on cEMA.

### Development of the Back End for Messaging and Data Management

Continuous engagement was crucial. We had to design a real-time ability into our app and its back end to enable our study team to see how participants were responding to the app and to give us the opportunity to reach out to participants who might be in danger of leaving the study. We used Amazon Web Services (AWS) to support actions based on participants’ responses or their relative time within the study. We chose to use AWS step functions and lambda functions to enable data to be placed within a DynamoDB structure and then processed by queries against a PostgreSQL database and with resulting values for display placed on S3. The step functions supported us in designing a logical series of actions based on a timer associated with when they signed up as a participant in the study.

Lastly, we gamified the participant’s actions within the app, to encourage their continued involvement. Tactics included virtual trophies for different stages of completion and a real-time leaderboard that displayed where each anonymous individual stood relative to others contributing to the project. Our goal was to encourage a feeling among participants that they were contributing to biomedical research, that it was being logged, and that they could do still more, relative to their peers. The back end additionally was set up to send reminders via SMS, emails, and alerts to encourage a sense of belonging, along with value, and to support continued awareness of the need to enter data.

### Comparisons Between Daily24 and Survey Data

Survey data collected as part of the parent study at baseline allowed a comparison to eating and sleeping windows reported through Daily24 to explore the accuracy and comprehensiveness of data entered through the app. Daily24 events are best viewed as an approximate daily entry to the weekly, monthly, and yearly habits of the participant. This approach to analysis may not reflect those with highly variable schedules (for example, Monday is always quite different than Wednesday). However, it provides a unified framework for approaching the very large number of individual data lines for each user.

## Results

**Figure 5:**
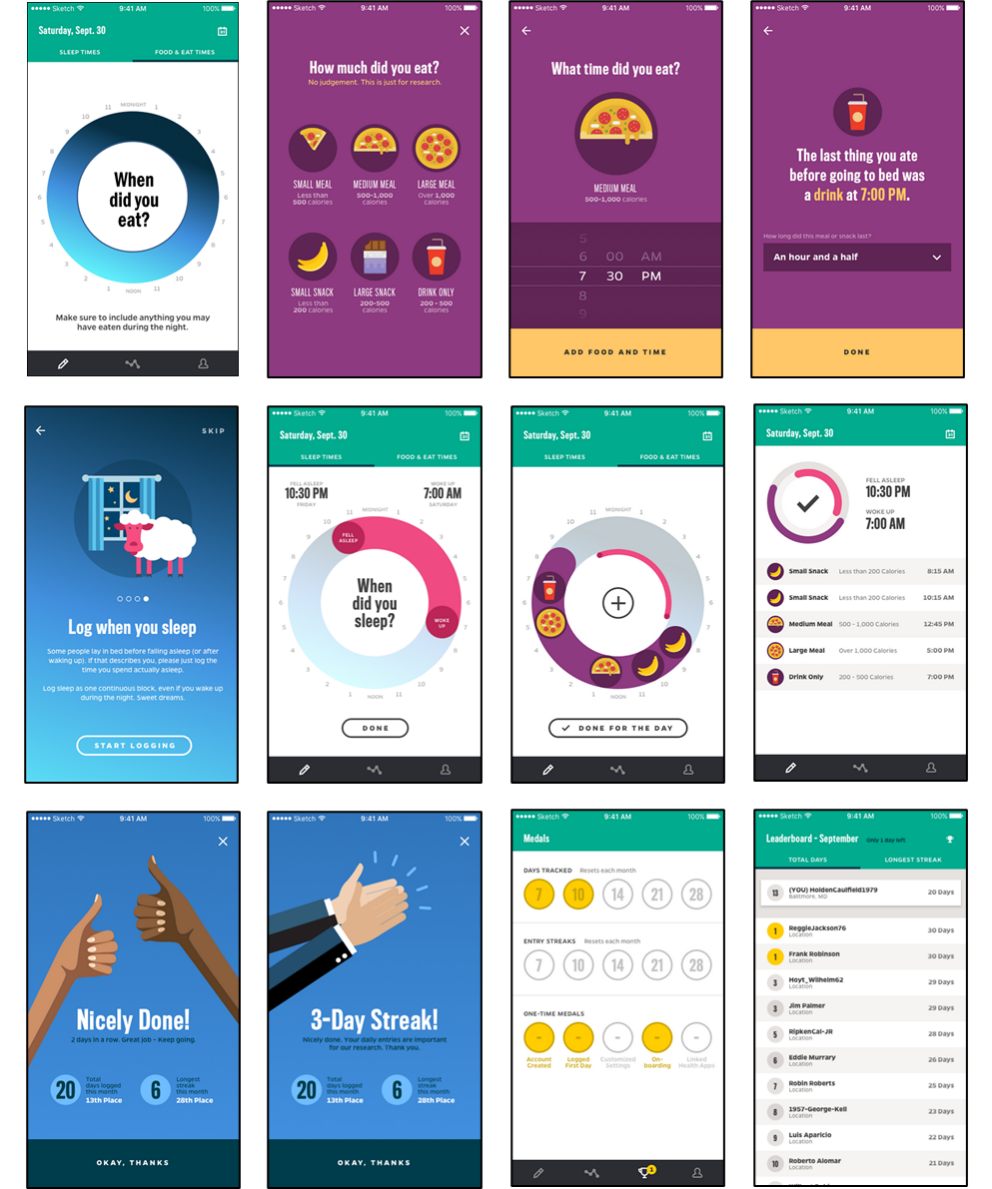
Daily24 mobile app screens.

The full application development process, including user testing and revisions, along with development of the back end, took approximately 2 years. The final requirements and decisions are summarized in Table 1, while the costs associated with each stage and product are detailed in Table 2. Figure 5 displays the final Daily24 mobile app screens.

**Table 1:** Requirements, decisions, and considerations in the design of Daily24, a circadian ecological momentary assessment (cEMA) app.

Requirement		Daily24 design decisions	Additional considerations
**Front end**			
	Collect recurring, circadian data, but limit collection to essential data	Focus on sleep and diet (and not activity)	Should be fast and easy to enter data; specific variables based on core (causal) model of the research hypothesis
	Rely on recurring user data entry	Sleep times, times of eating occasions, meal and snack sizes eaten	There are no passive entry approaches at this point in time
	Balance need for more research data against loss of engagement	Use categories of food size rather than specific foods and their calorie count	A simplified interface means that we gain from more daily interactions, but do lose some details
	Minimize labeling bias in data entry and minimize feedback	App does not communicate judgment	Avoid feeding back data (eg, summary graphs); avoid guiding users towards a particular change in behavior
	Maximize use over multiple days, minimize use within a day	Focus on data entry; simple interface	Target time frame was at least several weeks of daily input over 6 months
	Maximize user engagement	Gamify to encourage fun interactions with the app and to reward those that track leaderboards, virtual trophies, “Power28,” “PowerWeek”	A specific challenge for a data collection app that does not offer any direct benefits (as research is not supposed to)
	Maximize pool of potential research participants	Develop both for iOS and for Android; national + recruited	Used React Native to maximize developer time; trade off on national, beta-testing, commercial licensing, one-to-one distribution; national reviews might have decreased participation; loss of inter-app interoperability
	Maintain privacy	Nonidentifiable usernames selected using random word-pairing generator	Avoids concern about users creating identifiable usernames
	Manage expectations in comparison with commercial Apps (fitness trackers)	Address limited functionality up front during signup	None
	Balance research-data feedback among research needs, user expectations, and research ethics	N/A^a^	Users of commercial apps expect feedback; research argues against feedback; ethics argues in favor of research
	Involve target users in design	Stakeholder advisory board and user testing, iteration over options and discussions on what to take out and to leave in	None
	Support multiple types of users	“Super users” that want to enter multiple times each day vs “once-a-day” interaction for a short window of time	None
**Back end**			
	Minimize high-touch engagement costs	Real-time dashboards (updated hourly) and SMS and email messages	None
	Minimize development cost	Re-used our initial backend for Metabolic Compass and added dashboards	As an academic use case, revenue generation is not expected, and therefore development costs are not recovered
	Support the research process: Recruitment, Execution, Data Analysis	N/A	(Differs from commercial)
	Protect participant privacy	Designed in strongly from the beginning: used a UUID^b^ to link study data with messaging and created random usernames	HIPAA^c^ and IRB^d^ are major drivers of this desideratum
	Support multi-institutional recruitment and data collection	Designed in strongly from the beginning	We collected REDCap survey data and recruited from multiple institutions
	Continuous debugging and improvement of user experience	Used InstaBug to collect user feedback as well as technical issues	None

^a^N/A: not applicable.

^b^UUID: universally unique identifier.

^c^HIPAA: Health Insurance Portability and Accountability Act.

^d^IRB: institutional review board.

**Table 2:** Time to develop Daily24 as an example circadian ecological momentary assessment (cEMA) app.

Programming or study need		Personnel involved	Time estimate
**Front end**			
	Initial design workshop and planning	Study PI^a^, study co-PI, project coordinator, designer, summer student	1-2 months
	Design and stakeholder feedback	Designer, summer student, project coordinator, study PI, study co-PI	3-5 months
	Initial MVP^b^ rollout	Study coordinator, front end developer part-time, masters level part-time	6-9 months
	Iteration for design changes and bug tracking	Front end developer, study coordinator	3-4 months
	Near final with more bug reporting and testing	Front end developer, study coordinator	3-4 months
	Released version with bug tracking and iterations	Study coordinator, front end developer part-time	12 months or more
	AWS^c^ or other cloud provider charges	May also include Github, Instabug, and other expenses	12 months or more
**Back end: real-time dashboards and automated reminders to participants**			
	Lambda functions for analysis of incoming data	Back-end developer, masters-level part-time	1-2 months
	SMS via AWS step functions	Back-end developer	1-2 months
	Emails via AWS step functions	Back-end developer	1-2 months
	Initial design of dashboard	Study coordinator, back-end developer	1-2 months
	Iterative design to identify participants at risk of dropping out of study	Study coordinator, back-end developer	6 months or more
	AWS or other cloud provider charges	Will vary with usage	12 months or more
**Back end: datastores and analysis**			
	PostgreSQL tables	Back-end developer	3-5 months
	DynamoDB table	Back-end developer	3-5 months
	Trophy feedback	Back-end developer	1-2 months
	Leaderboard feedback	Back-end developer	1-2 months
	S3 backups and storage	Back-end developer	1-2 months
	Maintaining back end for blacklist updates	Bachelors level part-time	12 months or more
	SQL queries for analysis	Masters level part-time	12 months or more
	AWS or other cloud provider charges	Partially fixed, but then increases with more data	12 months or more

^a^PI: principal investigator.

^b^MVP: minimum viable product (meaning that it is functional, but only minimally).

^c^AWS: Amazon Web Services.

### User Testing Recommendations

We enrolled 37 test users through the qualitative study (mean age, 46.1 years; 26/37, 70% female; 19/37, 51% White) in addition to the 8-member advisory board. Overall, the advisory board and qualitative study participants agreed that the app was fast and easy to use, that the 24-hour wheels were an appropriate way to display the data collected, and that they appreciated the broad meal and snack size categories over needing to enter specific foods consumed. However, several participants identified bugs in the sleep wheel and calendar features, which demonstrated that the app was operating differently on different operating systems and phone models despite using React Native. The extra users were instrumental in identifying these differences and addressing the problems.

The advisory board highlighted a major area of concern: the best way to visually represent the meal and snack size options. The initial icons depicted different foods at the various size options. Advisory board members expressed a desire to select the icon that represented the type of food they had eaten rather than the size of the meal or snack. The icons were thus revised to show different portions of the same food (pizza pie) as a simple solution to address this concern (Figure 5). Advisory board members also shared ideas to make the calendar feature more visually engaging. Though they expressed a preference for entering interrupted sleep rather than a single sleep window, this feature was beyond the scope of this version of the app. They additionally identified bugs in the app and provided thoughts on the structure of prompts or reminders to enter data.

Qualitative study participants identified 2 main areas of concern in the design of the app. First, several users commented on their inability to record both a meal and a drink occurring at the same time, making it clear to the study team that the app did not clearly convey that the meal and snack icons encompassed drinks as well and that the drink only icon was to be used only in instances where a drink was consumed without food. The icon labels were reworded to more clearly convey this meaning, and the revised wording was tested with the remaining participants. A secondary concern regarding the inability to enter multiple sleep windows to capture interrupted night sleep or naps was also raised, as with the advisory board. The team used this feedback to clarify the app instructions to make it clear that only a single sleep window was to be entered.

App usage feedback highlighted the differences in usage patterns, with a split between participants who used the app throughout the day and those who used the app to enter all eating occasions one time per day. When asked to reflect on the times they did not use the app, participants were close to unanimous in requesting in-app reminders. They differed in their frequency preference, with some requesting multiple reminders per day to align with typical mealtimes and others requesting reminders only when a day passed without them entering data. This feedback was critical in the team’s design of the app reminders to be useful without irritating users who wanted less frequent reminders.

In addition, we elicited, through the advisory board and our qualitative study, what would motivate a long-term, 6-month research engagement with the app and how to structure that time. We expected that 6 months of continuous entry would be too burdensome for many users, particularly across our hoped-for population of 500 or more participants. We considered as a study team the optimal schedule for collecting data that spanned a 6-month period, taking into consideration the need to not overburden participants but to also not go so long between uses that re-engagement would be difficult, and shared ideas with the qualitative study participants for feedback. The final decision to use the Power28 and 5 PowerWeeks resulted from these discussions.

### Parent-Study Daily24 Usage Patterns

Our app met the goal of multiple, short sessions daily, with 70% of interactions with the app lasting less than 1 minute and with most users interacting with the app approximately 1 to 3 times during each day that they entered data. We found that roughly 25% of individuals used the app at least twice per day on days they entered data.

### Comparisons Between Daily24 and Survey Data

**Figure 6:**
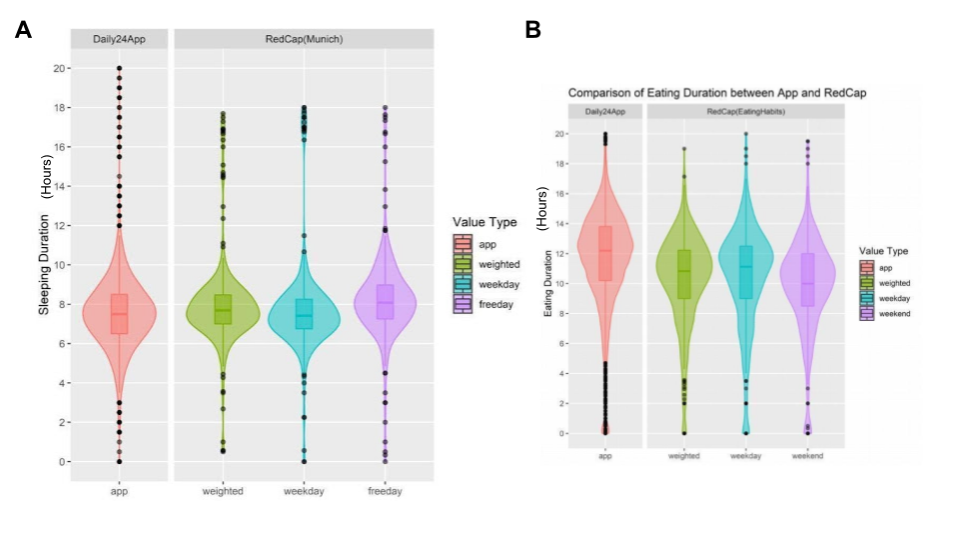
Comparison of (A) sleeping duration and (B) eating duration reported through the Daily24 mobile app and online survey.

**Figure 7:**
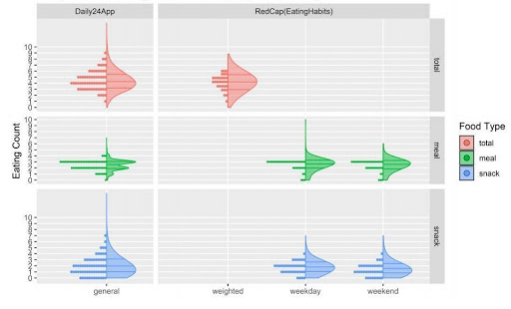
Comparison of eating counts reported through the Daily24 mobile app and the online survey.

Overall, the median values for sleep duration using the 2 modalities (Figure 6) are consistent, though there is more variability captured through the app data. The median length of the eating window reported through Daily24 is approximately 1 hour longer than that reported through a one-time survey recall, and the tail extends an additional hour. Similarly, the comparison between meal and snacks counts during the day (Figure 7) indicates that Daily24 captured more variability throughout the study period.

## Discussion

Overwhelmingly, we heard from users that entering data into the app was extremely efficient, presumably faster than other data collection platforms, and the app was obviously tailored for use on smartphones, which is often not the case for other data collection methods. Additionally, by the careful design of our reminders and by structuring our expectations for logging, we found that engaging and retaining participants without personal contact was possible. However, there were expectedly many bugs in the app that were difficult for our single programmer (during the support stage) to address, and users who encountered these bugs were frustrated that the app did not function as smoothly as commercial apps even after our attempts to mitigate their expectations.

The research context and goal of this type of app bring multiple challenges that are not present in the commercial realm. For example, there is no expectation for reclaiming any development costs when moving to production. At the outset, we recognized that users will be comparing the final product with commercial products from commercial development teams with much greater resources and larger engineering teams. Consequently, we designed our app with a tight range of objectives, a limited budget, and for a very special purpose. Others taking on this type of development should think carefully about the costs for design, development, testing, and continued production and analysis costs.

Though we attempted to communicate, through our recruitment materials and onboarding process, that this is a data collection app and not an app for losing weight, and further, that as a research app, this app would not have the features associated with commercially available apps, we found this message did not land with many participants. This most likely reflects the fact that most apps used daily are of extremely high quality and are supported by very large teams of programmers, data scientists, graphic artists, and advertising (or psychology-based) experts. Furthermore, as a health app associated with a renowned medical institution, users may have been predisposed to assume a health benefit of using the app. In retrospect, we needed to be clearer in our messaging to potential users of what they could — and could not — expect to gain personally from using the app and the difference between research and commercial apps to enable our participants to feel comfortable, rather than critical, at an early stage. Furthermore, we used our small incentives budget to purchase gift card prizes for weekly raffles, but in the future would try to find a larger incentives budget to compensate all participants who reach data entry milestones to provide additional incentive to use the app.

The value of the added accuracy and variability captured through the app depends on the goals of the individual project. Survey data reflect a perception of average daily behavior across many days or weeks and, as shown in the comparison of survey and app data, did not capture as much variability during the week or over a longer time span. For some studies, the extra effort and high cost to capture that additional variability may not be worth it, or perhaps other EMA approaches such as collecting data through instant messaging would capture sufficient variability at significantly lower cost. However, other projects might find that the story lies within that captured variability and may find the costs worthwhile.

The potential for the app to evolve for use in other studies may additionally factor into the cost-benefit analysis. The long-term benefits of app development become more apparent in projects that aim to build off initial results to study other populations or to develop behavior modification interventions. Several adjustments that the Daily24 architecture could accommodate are evident. For instance, the system of reminders and game rewards can be repurposed to support a shorter eating window. It would be easy for a participant to choose a time window for eating and then from that choice, both front end and back end could be working together to help reinforce that behavior. If a participant elected to start meals at a particular hour in the morning, a reminder could prompt them to enter whether they met that goal and to adjust their time entry for when they started their first meal. In a similar way, a prompt could readily be included to ask whether a participant has ended their meals for the day based on a time-based window for eating. The participant could then adjust the window to be smaller or larger based on their behavior. Game-like rewards for adhering to a time-based schedule would be easy to adjust from the current reward system for logging. This ability to adjust the app from a purely observational mode to a tool for behavior adjustment is a major benefit of the time and expense invested into the app.

Our experience is valuable for others considering cEMA apps and their associated back ends for clinical trials. We found that the development of an app and its associated back end was challenging, expensive, yet fully achievable in an academic setting. Like other research endeavors, we found that we could meet our major goals by carefully and iteratively identifying the data that we had to have and data or interactions we could live without. Our experience highlights the importance of a diverse and talented team, regardless of actual size, with an ability to be flexible and to listen to the participants. We believe that the future of this type of observational (and eventual interventional) study is with apps that encourage feedback from the participants, that enable the research team (often small and time-limited) to identify the participants most at risk of dropping out, and with an ability to collect, process, and display (subsets for the participants and all for the study team) real-time data. However, we suggest that anyone budgeting for this type of app be prepared for the long-term costs and the ups and downs associated with development and debugging and to expect that not all participants will “love the app.” 

## Abbreviations

AWS: Amazon Web Services
cEMA: circadian EMA
EMA: ecological momentary assessment
EMR: electronic medical record
